# 25-hydroxyvitamin D Levels and chronic kidney disease in the AusDiab (Australian Diabetes, Obesity and Lifestyle) study

**DOI:** 10.1186/1471-2369-13-55

**Published:** 2012-07-03

**Authors:** Matthew J Damasiewicz, Dianna J Magliano, Robin M Daly, Claudia Gagnon, Zhong X Lu, Peter R Ebeling, Steven J Chadban, Robert C Atkins, Peter G Kerr, Jonathan E Shaw, Kevan R Polkinghorne

**Affiliations:** 1 Department of Nephrology, Monash Medical Centre, 246 Clayton Road, Clayton, 3168, Victoria, Australia; 2 Monash University, Wellington Rd, Clayton, 3168, Victoria, Australia; 3 Baker IDI Heart and Diabetes Institute, 75 Commerical Rd, Melbourne, 3004, Victoria, Australia; 4 School of Exercise and Nutrition Sciences, Deakin University, Burwood Hwy, Burwood, 3125, Victoria, Australia; 5 Centre de recherche du CHUQ, 2325, rue de l'Université, Québec (Québec), Quebec G1V, 0A6, Canada; 6 Melbourne Pathology, 103 Victoria Parade, Collingwood, 3066, Victoria, Australia; 7 NorthWest Academic Centre, University of Melbourne, Western Health, 176 Furlong Road, St Albans, 3021, Victoria, Australia; 8 Department of Nephrology and Transplantation, Royal Prince Alfred Hospital, Missenden Road, Camperdown, 2005, NSW, Australia; 9 Sydney Medical School, University of Sydney, Sydney, 2006, NSW, Australia

**Keywords:** Albuminuria, Chronic kidney disease, Glomerular filtration rate, and Vitamin D

## Abstract

**Background:**

Low 25-hydroxy vitamin D (25(OH)D) levels have been associated with an increased risk of albuminuria, however an association with glomerular filtration rate (GFR) is not clear. We explored the relationship between 25(OH)D levels and prevalent chronic kidney disease (CKD), albuminuria and impaired GFR, in a national, population-based cohort of Australian adults (AusDiab Study).

**Methods:**

10,732 adults ≥25 years of age participating in the baseline survey of the AusDiab study (1999–2000) were included. The GFR was estimated using an enzymatic creatinine assay and the CKD-EPI equation, with CKD defined as eGFR <60 ml/min/1.73 m^2^. Albuminuria was defined as a spot urine albumin to creatinine ratio (ACR) of ≥2.5 mg/mmol for men and ≥3.5 for women. Serum 25(OH)D levels of <50 nmol/L were considered vitamin D deficient. The associations between 25(OH)D level, albuminuria and impaired eGFR were estimated using multivariate regression models.

**Results:**

30.7% of the study population had a 25(OH)D level <50 nmol/L (95% CI 25.6-35.8). 25(OH)D deficiency was significantly associated with an impaired eGFR in the univariate model (OR 1.52, 95% CI 1.07-2.17), but not in the multivariate model (OR 0.95, 95% CI 0.67-1.35). 25(OH)D deficiency was significantly associated with albuminuria in the univariate (OR 2.05, 95% CI 1.58-2.67) and multivariate models (OR 1.54, 95% CI 1.14-2.07).

**Conclusions:**

Vitamin D deficiency is common in this population, and 25(OH)D levels of <50 nmol/L were independently associated with albuminuria, but not with impaired eGFR. These associations warrant further exploration in prospective and interventional studies.

## Background

Vitamin D plays an important role in human health, and vitamin D deficiency is commonly observed in the general population and across the spectrum of chronic kidney disease (CKD) [1]. Traditionally, vitamin D has been linked to bone and mineral metabolism, however increasingly it has been also recognised to play a pivotal role in the physiology of numerous other organs including the endocrine, cardiovascular and immune systems [2].

CKD affects between 10-15% of the adult population[3]. The decline in renal function is paralleled by a reduction in calcitriol synthesis, and calcitriol replacement has been associated with improved mortality in patients with CKD [4-7]. Many extra-renal cells are able to convert the circulating form of vitamin D, 25-hydroxy vitamin D (25(OH)D), into the active form [8]. This has focused research on the autocrine function of vitamin D, and serum 25(OH)D deficiency has emerged as an important independent risk factor for morbidity and mortality, and a potential therapeutic target in CKD.

Experimental data show that vitamin D is a potent inhibitor of the renin-angiotensin system (RAS) [9] and nuclear factor (NF)-kB pathway [10], and in human studies low vitamin D levels have been independently associated with higher plasma renin and angiotensin 2 concentrations [11]. These pathways play an important role in the pathogenic processes of kidney disease, mediating immune, inflammatory and proliferative effects that culminate in progressive renal damage [12]. 25(OH)D deficiency may potentiate the decline in renal function as well as the progression of albuminuria, which is in itself an established predictor of CKD progression and cardiovascular outcomes [13-15].

In general population studies such as the Third National Health and Nutrition Examination Survey (NHANES III), 25(OH)D deficiency has been associated with prevalent albuminuria [16] and progression to end-stage kidney disease (ESKD) [17]. In a population aged 65 years and older, 25(OH)D deficiency was associated with glomerular filtration rate (GFR) loss [18]. In the CKD population 25(OH)D deficiency has also been associated with prevalent albuminuria [19], progression to ESKD [20] and mortality [21]. In a recent meta-analysis higher 25(OH)D levels were associated with improved survival [22].

In a large, general population cohort of adults aged 25 years and over participating in the AusDiab study, we examine the cross-sectional association of 25(OH)D levels with albuminuria and impaired GFR. Existing literature supports an association between 25(OH)D and albuminuria, however its association with renal function is less clear. We hypothesized that 25(OH)D deficiency would be highly prevalent and associated with CKD, however this association is likely to be stronger with albuminuria rather than decreased eGFR.

## Methods

### Study population

AusDiab was a population-based survey of adults aged 25 years and older, which used cross-sectional data collected at baseline (1999–2000). Details of the survey methods and sample collection have been previously described in detail [23]. In brief, a sample of the population was obtained using a stratified sampling cluster method. Of 17,129 households eligible for inclusion, 11,479 agreed to be interviewed and 20,293 adults completed the household interview. A total of 11,247 (55.3%) adults presented for the physical examination. A complete data set was available for 10,732 (95.4%), and was used in all analyses. The overall response rate for the study was approximately 37%. The AusDiab study was approved by the International Diabetes Institute ethics committee (Melbourne, Australia), and written informed consent was obtained from all participants. The consent included permission for any data obtained at the time of the baseline analysis to be used in future studies.

### Study measurements

Details of the biomedical tests have been previously reported [23,24]. In brief, those who attended a testing site at baseline underwent a physical examination, which consisted of a fasting blood sample, a random spot urine morning collection and a standard 75-g oral glucose tolerance test. Demographic data and information were collected using standardized interviewer-administered questionnaires.

Hypertension was defined as a systolic blood pressure of 140 mmHg or greater or a diastolic blood pressure of 90 mmHg or greater. Body mass index (BMI) was calculated from weight and height measurements, with the diagnosis of obesity defined as a body mass index of 30 kg/m^2^ or greater. Standard World Health Organization criteria for the diagnosis of abnormal glucose metabolism were used. Diabetes was diagnosed on the basis of fasting plasma glucose level of 7.0 mmol/L (126.1 mg/dl) or greater, 2-hour plasma glucose level of 11.1 mmol/L (200 mg/dl) or greater, or current treatment with insulin or oral hypoglycemic medication. Smoking status was self-reported, and subjects were classified as either current smoking or non-smoking (ex-smokers and never smoked). Cholesterol and triglyceride levels were measured on fasting serum samples, and assessed as continuous variables. Cardiovascular disease was assessed using self-reported symptoms or past history. Ethnicity was categorized into “Europid” and “non-Europid” based on the country of birth. The majority of participants were classified as “Europid”, which included those born in Australia, New Zealand, North America and Northern Europe. Time of assessment was classified by the season in which venipuncture was performed. The location of the subjects (urban versus rural) was based upon the classification used by the Australian Bureau of Statistics. The latitude of blood collection centers was determined using the Google® GPS tool and entered as a continuous variable for analysis (range 12°S to 43°S). Total leisure-time physical activity reported for the previous week (none, insufficient: 1–149 min; sufficient: ≥150 min) was measured using the Active Australia questionnaire, which is the standard instrument for population surveillance and has acceptable reliability [25].

### Laboratory methods

Serum 25(OH)D was measured in the entire baseline AusDiab cohort from samples, which were stored at −80°C. A direct competitive chemiluminescent immunoassay (CLIA) was performed in our laboratory using a Liaison analyser (DiaSorin Inc., Stillwater, MN, USA) with an inter-assay CV of 7.0% at 45 nmol/L and 6.3% at 93 nmol/L. For participants where fasting serum was not available (n = 210), fluoride oxalate plasma was used. To evaluate whether fluoride oxalate plasma was comparable with fasting serum samples for the 25(OH)D analysis, we analysed both simultaneously from samples collected from 101 laboratory staff. There was excellent agreement between the two tubes: fluoride oxalate plasma 25(OH)D = 0.97 x serum 25(OH)D + 2.5, *r² =* 0.89 [26].

Serum creatinine was reassessed on all baseline samples using an IDMS aligned enzymatic method (Roche Modular, Roche Diagnostics, Indianapolis IN). Urine albumin was measured by means of rate nephelometry with the Beckman Array (Beckman/Coulter, Fullerton, USA) using fresh urine samples at the time of original collection. Urine creatinine was measured using the modified kinetic Jaffé reaction using an Olympus AU600 autoanalyzer (Olympus Optical Co. Ltd., Tokyo, Japan).

### Study outcomes

Vitamin D deficiency was defined as a serum 25(OH)D level of <50 nmol/L [27,28]. For subgroup analysis, common clinical cut-points were used, with 25(OH)D levels of <75, <50 and <25, classified as insufficient, deficient and severely deficient. Microalbuminuria was defined as a urine albumin-creatinine ratio (ACR) of ≥2.5 mg/mmol in males and ≥3.5 in females, with macroalbuminuria defined as a urine ACR of ≥25 mg/mmol [29]. GFR was estimated using the Chronic Kidney Disease epidemiology (CKD-EPI) equation for white men and women[30]. Impaired GFR was defined as an eGFR <60 ml/min/1.73 m^2^[31].

### Statistical analysis

Analyses were weighted to represent the non-institutionalized Australian population, accounting for non-response and the stratified cluster sampling method of the survey design to produce nationally representative estimates. All estimated means, proportions, odds ratios, and their standard errors, were adjusted for the survey design by using the survey commands for analyzing complex survey data. Urine ACR was natural log transformed because of the skewed nature of the data.

Logistic regression was used to assess the relationship between the two separate CKD variables (albuminuria and impaired eGFR) and the odds of vitamin D deficiency. Three models were performed: unadjusted, adjusted for gender and age, and fully adjusted. Covariates examined included age, gender, BMI, cholesterol, triglycerides, cardiovascular disease, smoking status, ethnicity, diabetic status, location (urban or rural), latitude, season, exercise, ethnicity and blood pressure. The albuminuria models were also adjusted for eGFR, and the eGFR models were adjusted for albuminuria. The fully adjusted multivariate model was constructed including covariates if they were clinically important or confounders. Finally we used multinomial logistic regression to explore the relationship between levels and severity of albuminuria and eGFR and vitamin D deficiency. Interactions between the CKD outcomes and age, sex, diabetes mellitus were also assessed. All analyses were conducted using Stata/IC, version 11.1 (Stata Corp, College Station, TX).

## Results

Table 1 summarises the baseline characteristics of the total population, and stratified by 25(OH)D deficiency. 10,732 participants in the baseline survey were included in the final analysis. The mean age of the subjects was 48.2 years (95% CI 46.5-49.9).

**Table 1:** Characteristic of the cohort by vitamin D deficiency

	**25(OH) vitamin D level nmol/L**			
**Characteristic**	**Total (n = 10732)**	**≥ 50 (n = 7622)**	**< 50 (n = 3110)**	**p**
Age (mean, [SE])	48.2 [0.82]	46.8 [0.92]	51.3 [0.68]	<0.001
**Age group**				
25–44 (%, [SE])	47.5 [2.48]	51.3 [2.80]	39.0 [1.94]	
45–64 (%, [SE])	33.9 [1.85]	32.1 [2.11]	37.8 [1.61]	
≥65 (%, [SE])	18.6 [1.80]	16.6 [1.93]	23.2 [1.85]	<0.001
Female (% [SE])	49.9 [0.57]	44.0 [0.70]	63.8 [1.71]	<0.001
Europid (% [SE])	87.3 [2.74]	92.1 [1.76]	76.6 [4.48]	<0.001
Diabetes (% [SE])	7.0 [0.66]	5.1 [0.49]	11.1 [1.04]	<0.001
Hypertension (% [SE])	22.5 [1.45]	20.0 [1.74]	28.0 [0.97]	<0.001
Smoking (% [SE])	16.4 [1.77]	16.4 [1.72]	16.4 [2.72]	0.98
**Current**				
History of CVD (% [SE])	6.1 [0.57]	5.1 [0.52]	8.2 [0.80]	<0.001
Season (% [SE])				
summer (Dec –Feb)	12.8 [6.72]	16.3 [8.27]	4.9 [2.72]	<0.001
autumn (Mar –May)	14.2 [5.10]	13.3 [4.81]	16.2 [6.04]	
winter (Jun –Aug)	26.7 [5.56]	23.3 [5.22]	34.3 [7.31]	
spring (Sep –Nov)	46.3 [7.07]	47.1 [7.86]	44.7 [6.56]	
**BMI (% [SE])**				
25–29.9	39.2 [0.56]	40.7 [0.78]	36.1 [1.28]	<0.001
≥ 30	20.3 [1.05]	17.3 [1.17]	27.0 [1.60]	
Total cholesterol (mean [SE])	5.54 [0.03]	5.43 [0.03]	5.79 [0.03]	<0.001
Location (%, [SE]) Urban	57.0 [10.5]	53.8 [10.7]	64.1 [11.0]	0.11
Albuminuria (%, [SE])	6.9 [0.60]	5.4 [0.52]	10.5 [1.05]	<0.001
eGFR < 60 (%, [SE])	2.7 [0.28]	2.3 [0.30]	3.5 [0.47]	0.03

SE – standard error, CVD - cardiovascular disease, BMI – body mass index, eGFR – estimated glomerular filtration rate.

The mean 25(OH)D level was 62.8 nmol/L (95% CI 58.8-66.8). The proportion of study participants with a 25(OH)D level of <25, 25–49, 50–74 and ≥75 nmol/L was 4.1%, 26.6%, 42.1% and 27.2%, respectively. Mean 25(OH)D levels were lower in females (57.9 versus 67.7 nmol/L, *p* < 0.001) and non-Europids (47.1 versus 65.1 nmol/L, *p* < 0.001). The mean 25(OH)D levels in those aged 25–44, 45–64 and >65 years were 67.1 (95% CI, 61.6-72.6), 59.8 (95% CI 56.9-62.6) and 57.3 (95% CI 54.1-60.4) nmol/L (*p* trend <0.001), respectively. 25(OH)D deficiency was more prevalent with increasing age, female gender, non-European background, medical co-morbidities (diabetes, hypertension, cardiovascular disease, obesity) and the presence of albuminuria and an impaired GFR (Table 1).

### Impaired eGFR

The mean eGFR in the study cohort was 99.1 ml/min/1.73 m^2^ (95% CI, 97.7-100.6). The proportion of study participants with an eGFR of <60 ml/min/1.73 m^2^, was 2.7% (95% CI 2.1-3.3). Of these participants, the proportion with CKD stage 3a, 3b, and 4/5 was 70.5%, 19.4%, and 10.2%, respectively. The mean 25(OH)D levels were lower in those with an eGFR of <60, (57.2 versus 63.0 nmol/L, *p* = 0.02). 25(OH)D deficiency was significantly associated with an eGFR <60 ml/min/1.73 m^2^ in the unadjusted model (OR 1.52, 95% CI 1.07-2.17, *p* = 0.02). However the association was attenuated and no longer significant when adjusted for age and gender (OR 0.96, 95% CI, 0.71-1.30), *p* = 0.78), and in the fully adjusted model (OR 0.95, 95% CI, 0.67-1.35, *p* = 0.78).

The relationship between vitamin D level and eGFR was further explored with the study population being divided into quartiles based upon the 25(OH)D and population cut-off levels (Table 2). 25(OH)D levels in the lowest quartile (OR 0.73, 95% CI 0.43-1.25, *p* trend 0.53) or severe 25(OH)D deficiency (OR 0.93, 95% CI 0.35-2.51, *p* trend 0.46) were not significantly associated with an impaired eGFR. No interactions were found on the impaired eGFR and vitamin D deficiency relationship by gender, age or diabetes mellitus (*p* > 0.10).

**Table 2:** Impaired eGFR regression models - vitamin D quartiles study population & population cut-off points

	**eGFR <60 OR (95% CI)**		
**25(OH)D level nmol/L**	**Model 1**^**a**^	**Model 2**^**b**^	**Model 3**^**c**^
**≥77**	Ref	Ref	Ref
**61-76**	0.87 (0.37-2.01)	0.57 (0.29-1.12)	0.56 (0.28-1.09)
**47-60**	1.11 (0.62-2.00)	0.69 (0.39-1.21)	0.69 (0.37-1.28)
**<47**	1.62 (0.90-2.91)	0.76 (0.49-1.19)	0.73 (0.43-1.25)
***P*trend**	0.05	0.57	0.53
	**eGFR <60 OR (95% CI)**		
**≥75**	Ref	Ref	Ref
**50-74**	1.02 (0.60-1.75)	0.67 (0.44-1.02)	0.65 (0.40-1.06)
**25-49**	1.45 (0.87-2.42)	0.73 (0.49-1.07)	0.68 (0.42-1.09)
**<25**	2.14 (0.80-5.73)	0.73 (0.29-1.80)	0.93 (0.35-2.51)
***P*trend**	0.08	0.33	0.46

* p < 0.05, ^#^p < 0.01, ^†^ p < 0.001.

OR - odds ratio, CI – confidence interval.

^a^ unadjusted.

^b^ adjusted for age and gender.

^c^ adjusted for age, gender, diabetes, body mass index, triglycerides, cardiovascular disease, smoking, season, albuminuria and systolic blood pressure.

Associations between 25(OH)D deficiency and severity of renal impairment were also examined (Table 3). 25(OH)D deficiency was significantly associated with an eGFR <30 in the univariate model (OR 9.31 95% CI 2.11-41.1, *p* = 0.004), however not upon multivariate analysis (OR 4.46, 95% CI 0.88-22.7, *p* = 0.07), when compared to those with an eGFR ≥60.

**Table 3:** Associations of 25(OH)D levels and severity of albuminuria and Impaired eGFR

	**25(OH)D < 50 nmol/L, OR (95% CI)**					
	**Model 1**^**a**^	**p**	**Model 2**^**b**^	**p**	**Model 3**^**c**^	**p**
**No Albuminuria**	Ref		Ref		Ref	
**Micro-albuminuria**	2.06 (1.51-2.82)	<0.001	1.80 (1.35-2.41)	<0.001	1.59 (1.15-2.21)	0.007
**Macro-albuminuria**	1.99 (0.95-4.17)	0.07	1.70 (0.92-3.13)	0.09	1.22 (0.76-1.98)	0.39
	**25(OH)D < 50 nmol/L, OR (95% CI)**					
	**Model 1**^**a**^		**Model 2**^**b**^		**Model 3**^**d**^	
**eGFR ≥60**	Ref		Ref		Ref	
**eGFR 30-59**	1.25 (0.78-2.02)	0.34	0.79 (0.53-1.16)	0.22	0.84 (0.56-1.26)	0.39
**eGFR <30**	9.31 (2.11-41.1)	0.004	5.93 (1.20-29.2)	0.03	4.46 (0.88-22.7)	0.07

CI – confidence interval, OR – odds ratio.

^a^ unadjusted.

^b^ adjusted for age and gender.

^c^ adjusted for age, gender, diabetes, body mass index, triglycerides, cardiovascular disease, smoking, season, glomerular filtration rate and systolic blood pressure.

^d^ adjusted for age, gender, diabetes, cholesterol, triglycerides, body mass index, smoking, ethnicity, cardiovascular disease, albuminuria and systolic blood pressure.

### Albuminuria

Albuminuria was present in 6.9% (95% CI 5.7-8.2) of study participants, with the majority, 85.7% having microalbuminuria. Participants with 25(OH)D deficiency were twice as likely to have albuminuria in the unadjusted model (OR 2.05, 95% CI 1.58-2.67, *p* < 0.001) and the association remained significant when adjusted for age and gender (OR 1.79, 95% CI 1.40-2.29), *p* < 0.001). In the fully adjusted model, subjects with 25(OH)D deficiency had a 54% increased likelihood of albuminuria compared to those without deficiency (OR 1.54, 95% CI 1.14-2.07, *p* = 0.006).

The effect of vitamin D level was further explored by dividing the study population into quartiles based on vitamin D levels (Table 4). Subjects in the quartile with the lowest 25(OH)D levels had an increased likelihood of albuminuria compared to those with higher 25(OH)D levels, in the unadjusted (OR 2.40, 95% CI 1.55-3.73, *p* trend <0.001) and fully adjusted (OR 1.48, 95% CI 0.96-2.27, *p* trend = 0.007) models. When the study population was divided into population cut-off levels, 25(OH)D levels of <25 nmol/L were significantly associated with albuminuria unadjusted OR (2.60, 95% CI, 1.55-3.73, *p* trend <0.001), but not fully adjusted OR (1.22, 95% CI 0.42-3.58, *p* trend 0.13). This may be explained by the small proportion of participants who were profoundly vitamin D deficient (n = 310, 2.89%). No interactions were found on the albuminuria and vitamin D deficiency relationship by gender, age and diabetes mellitus (all interaction *p* > 0.10).

**Table 4:** albuminuria regression models - vitamin D quartiles study population & population cut-off points

	**Albuminuria OR (95% CI)**		
**25(OH)D level nmol/L**	**Model 1**^**a**^	**Model 2**^**b**^	**Model 3**^**c**^
**≥77**	Ref	Ref	Ref
**61-76**	1.00 (0.66-1.53)	0.82 (0.55-1.22)	0.73 (0.48-1.11)
**47-60**	1.40 (0.93-2.10)	1.14 (0.80-1.62)	1.02 (0.72-1.44)
**<47**	2.40 (1.55-3.73)^†^	1.87 (1.26-2.76)^#^	1.48 (0.96-2.27)
***P*trend**	<0.001	<0.001	0.007
	**Albuminuria OR (95% CI)**		
**≥75**	Ref	Ref	Ref
**50-74**	1.15 (0.81-1.67)	0.96 (0.70-1.34)	0.86 (0.62-1.19)
**25-49**	2.20 (1.42-3.40)^#^	1.74 (1.18-2.56)^#^	1.42 (0.93-2.16)
**<25**	2.60 (1.02-6.64)^*^	1.84 (0.42-56.5)	1.22 (0.42-3.58)
***P*trend**	<0.001	0.005	0.13

*p < 0.05, ^#^p < 0.01, ^†^p < 0.001.

OR - odds ratio, CI – confidence interval.

^a^ unadjusted.

^b^ adjusted for age and gender.

^c^ adjusted for age, gender, diabetes, body mass index, triglycerides, cardiovascular disease, smoking, season, glomerular filtration rate and systolic blood pressure.

Associations between 25(OH)D deficiency and severity of albuminuria were also explored (Table 3). Upon multivariate analysis, vitamin D deficiency was associated with an increase in microalbuminuria (OR 1.59, 95% CI, 1.15-2.21, *p* = 0.007), but not macroalbuminuria (OR 1.22, 95% CI, 0.76-1.98, *p* = 0.39), when compared to those with no albuminuria. This may be explained by the small proportion of participants (n = 112, 0.9%) with macroalbuminuria.

## Discussion

In a large, general-population cohort, 25(OH)D deficiency was common and independently associated with prevalent albuminuria. The association between serum 25(OH)D and albuminuria was most pronounced in those with a 25(OH)D level in the lowest quartile (<47 nmol/L) and remained significant in the multivariate model. Our study did not demonstrate a significant association between 25(OH)D deficiency and an impaired eGFR.

The association between 25(OH)D deficiency and albuminuria was also described in the NHANES III cohort[16]. Both this study and ours are similar in that they are general population based surveys with a low prevalence of advanced CKD. Nevertheless the NHANES III cohort differs to our study population, with a lower prevalence of 25(OH)D deficiency (22.2% versus 30.7), a higher prevalence of albuminuria (11.6% versus 6.9%), and hypertension (30.9% versus 22.5%), and greater ethnic diversity (non-Caucasians, 54.1% versus 87.3), The consistency of this finding in two, large cohorts highlights the robust nature of this association. This association was also demonstrated in a CKD cohort from the SEEK (Study of Early Evaluation of Chronic Kidney Disease) study [19], where 25(OH)D deficiency was independently associated with prevalent albuminuria.

Plausible mechanistic links exist between serum 25(OH)D deficiency and albuminuria. Activation of the Wnt/β-catenin signaling pathway induces podocyte injury in animal models [32], and this pathway can be blocked by paricalcitol administration [33]. A number of experimental studies demonstrate the reno-protective effects of vitamin D, likely mediated through the RAS and NF-κB [34]. Mice lacking the vitamin D receptor or α-hydroxylase (required for vitamin D activation), constitutively over-express renin and develop hypertension [35]. In animal models of renal injury, vitamin D analogues have been shown to attenuate interstitial fibrosis [36], glomerulosclerosis [37] and CKD progression [38]. Vitamin D is known to decrease renin gene transcription[9], and administration of vitamin D analogues resulted in decreased renin expression and consequently reduced Angiotensin II expression [39]. As Angiotensin II is a key mediator of proteinuria and kidney damage through haemodynamic (vasoconstriction) and non-haemodynamic (cell proliferation, fibrosis, oxidative stress) means [40], this is one likely mechanism of the protection afforded by vitamin D.

NF-κB is involved in the regulation of inflammatory cytokines and may promote inflammation and fibrogenesis in experimental and clinical kidney disease [41]. Fibroblasts derived from mice lacking the vitamin D receptor have intrinsic activation of NF-κB [10]. This appears relevant to kidney disease as administration of paricalcitol to mice with experimental obstructive nephropathy was found to block NF-κB and attenuate tubule-interstitial inflammation [36]. Inflammatory cytokines such as transforming growth factor-β and monocyte chemo-attractant protein-1 have been implicated in the pathogenesis of nephropathy [42,43]. In a study of CKD patients, low levels of vitamin D metabolites were associated with increased inflammatory parameters [44].

Clinical studies have examined the effects of vitamin D on proteinuria in CKD. Calcitriol and paricalcitol administration reduced proteinuria in patients with IgA [45] and diabetic nephropathy [46], already on RAS blockade. In a recent study high dose cholecalciferol, showed a reduction in proteinuria in patients with diabetic nephropathy on RAS blockade [42]. The significant increase in serum calcitriol level observed also highlight the important role of paracrine calcitriol synthesis in mediating the “non-classical” effects of vitamin D. Interestingly proteinuria can also contribute to 25(OH)D deficiency through a decrease in the megalin-mediated reuptake of 25(OH)D in the proximal tubule [47], however this is only likely to be of clinical significance in cases of heavy proteinuria.

In our study cohort with a low prevalence of severe renal impairment, we did not demonstrate a significant relationship between serum 25(OH)D levels and impaired eGFR in the multivariate models, which is consistent with the NHANES III data [48]. Our study did show an association between 25(OH)D deficiency and an eGFR <30, however this was lost upon multivariate adjustment. Longitudinal studies demonstrate an association between 25(OH)D deficiency and progression to ESKD. In 13,328 participants from NHANES III, 25(OH)D levels <15 ng/ml (<38 nmol/L) were significantly more likely to progress to ESKD over a median follow-up of 9.1 years [17]. In 1705 participants aged 65 years and older, 25(OH)D <15 ng/ml were associated with more rapid GFR loss [18]. In a smaller study of mild-moderate CKD, low 25(OH)D levels were associated with increased likelihood of CKD progression [20].

The association between low serum 25(OH)D levels and CKD progression, but not prevalence of low eGFR may be important. It is clear that many among the general community with stage 3 CKD do not progress [49]. As low serum 25(OH)D is associated with albuminuria and renal inflammation – two key predictors of CKD progression, it may well be that low serum 25(OH)D may be a useful marker of risk progression amongst those with a lower eGFR. It is possible that GFR derived with new equations that use cystatin C alone, or in combination with creatinine, may allow for better stratification of patients [50], and help to delineate any potential associations. Given the generally slow rate of CKD progression, it is possible that longitudinal analysis may differentiate between stable and progressive CKD and better delineate this association.

The strengths of this study include the recruitment of a large, national, population-based cohort, a standardized interview and examination process, and all biochemical measurements being performed in a central laboratory. In contrast to previous population-based studies, the prevalence of impaired renal function is more likely to reflect clinically significant renal impairment given the use of the CKD-EPI equation and calibrated enzymatic serum creatinine. However, there are also several limitations. The cross-sectional design does not infer causality and the associations described may be confounded by unknown and unmeasured factors. The baseline 25(OH)D levels may not be an accurate reflection of lifetime 25(OH)D levels, however one recent study suggests that vitamin D status tends to remain stable over time [51]. Calcitriol levels and additional markers of mineral metabolism (phosphate, parathyroid hormone and fibroblast growth factor-23) were not measured and these may confound the relationship between 25(OH)D levels and CKD progression. Similarly biochemical markers of the RAS, such as plasma renin levels were also not recorded. Serum creatinine and albuminuria were recorded with a single measurement, introducing the potential for misclassification bias. Calcitriol use and vitamin D supplementation were not recorded. It is likely that calcitriol use would be negligible given the low prevalence of CKD and local prescribing guidelines. Similarly any prolonged use of vitamin D or multi-vitamin supplements should be reflected in the serum 25(OH)D levels. Medications known to affect CKD progression, in particular ACE-inhibitors and angiotensin receptor blockers were also not recorded. These medications are likely to confer a protective benefit over and above that of blood pressure control, and their use may therefore decrease any positive effect of vitamin D observed.

## Conclusions

Many questions regarding the role of vitamin D in CKD remain unanswered. The optimal 25(OH)D levels are not well established and may vary depending on the underlying disease state and population studied. It is also not clear how accurately serum 25(OH)D levels reflect local tissue concentrations, which is paramount given the importance of autocrine vitamin D synthesis in mediating its extra-renal effects and proposed benefits. Finally, the ideal way to replace vitamin D is still not clear, and this may also depend on the clinical situation. The exact role of vitamin D supplementation in CKD needs further evaluation given the traditional propensity of nephrologists to prescribe “active” vitamin D compounds.

In summary, our study demonstrates a strong association between 25(OH)D deficiency and albuminuria but not impaired eGFR. Given the associations described, taken together with the limited clinical data and more compelling experimental data demonstrating kidney protective effects of vitamin D, it is tempting to speculate that the correction of vitamin D deficiency may present a novel, strategy to delay CKD progression. These results need prospective evaluation and validation in adequately powered, interventional trials examining the effect of vitamin D on the reduction of proteinuria. More importantly, these studies will need to demonstrate a reduction in meaningful clinical end-points such as a decrease in ESKD and mortality.

## Abbreviations

25(OH)D: 25-hydroxyvitamin D; GFR: Glomerular filtration rate; CKD: Chronic kidney disease; CKD-EPI: Chronic kidney disease epidemiology collaboration; eGFR: Estimated glomerular filtration rate; ACR: Albumin to creatinine ratio; OR, Odds ratio; CI: Confidence interval; RAS: Renin angiotensin system; NHANES: National health and nutrition examination survey; ESKD: End-stage kidney disease; CLIA: Chemiluminescent immunoassay.

## Competing interests

The authors’ have no competing interests to declare.

## Authors’ contributions

MJD; main statistical analysis and manuscript preparation, contribution to study design, interpretation of data. DJM; manuscript preparation and contribution to study design, interpretation of data. RMD; manuscript preparation and contribution to study design, interpretation of data. CG; manuscript preparation and contribution to study design, interpretation of data. ZXL; manuscript preparation and contribution to study design, biochemical analysis of vitamin D and serum Cr, interpretation of data. PRE; manuscript preparation and contribution to study design, interpretation of data. SJC; manuscript preparation and contribution to study design, interpretation of data. RCA; manuscript preparation and contribution to study design, interpretation of data. PGK; manuscript preparation and contribution to study design, interpretation of data. JES; manuscript preparation and contribution to study design, interpretation of data. KRP: statistical analysis and manuscript preparation, contribution to study design, interpretation of data. All authors read and approved the final manuscript.

## Pre-publication history

The pre-publication history for this paper can be accessed here:

http://www.biomedcentral.com/1471-2369/13/55/prepub
